# Correction: Novel biodegradable and non-fouling systems for controlled-release based on poly(ε-caprolactone)/Quercetin blends and biomimetic bacterial S-layer coatings

**DOI:** 10.1039/c9ra90063b

**Published:** 2019-08-27

**Authors:** Eva Sanchez-Rexach, Jagoba Iturri, Jorge Fernandez, Emilio Meaurio, Jose-Luis Toca-Herrera, Jose-Ramon Sarasua

**Affiliations:** Department of Mining-Metallurgy Engineering and Materials Science, University of the Basque Country UPV/EHU Plaza Ingeniero Torres Quevedo 1 Bilbao 48013 Spain evagloria.sanchez@ehu.eus; Institute for Biophysics, Department of Nanobiotechnology, University of Natural Resources and Life Sciences (BOKU) Muthgasse 11 (Simon Zeisel Haus) Vienna 1190 Austria

## Abstract

Correction for ‘Novel biodegradable and non-fouling systems for controlled-release based on poly(ε-caprolactone)/Quercetin blends and biomimetic bacterial S-layer coatings’ by Eva Sanchez-Rexach *et al.*, *RSC Adv.*, 2019, **9**, 24154–24163.

The authors regret that Fig. 7 was not displayed correctly in the original article. It should appear as presented below.

**Fig. 7:**
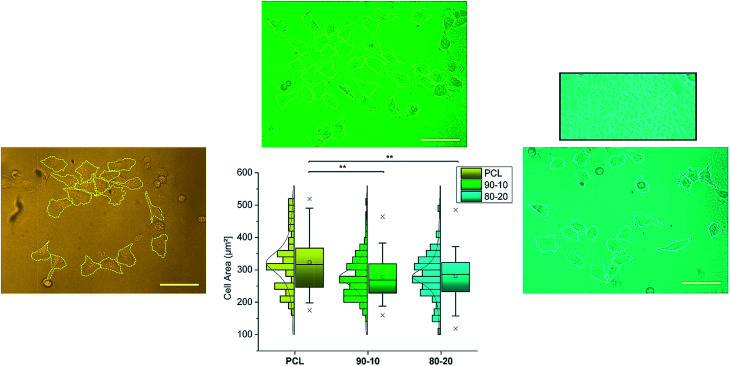
Cell area calculation from the corresponding optical micrographs on the different PCL systems. Statistical analysis was performed for *N* > 100 and a one-way ANOVA determined the significance (*p* < 0.01, represented by “**”) of the recorded variations.

The Royal Society of Chemistry apologises for these errors and any consequent inconvenience to authors and readers.

## Supplementary Material

